# Supplementary material for: Regulation of ER Composition and Extent, and Putative Action in Protein Networks by ER/NE Protein TMEM147
Source: Int J Mol Sci. 2021 Sep 23;22(19):10231. doi: 10.3390/ijms221910231 (PMC8508377; doi:10.3390/ijms221910231)
Supplement: Supplementary file 1 [file ijms-22-10231-s001.zip › ijms-1384055-supplementary.pdf]

International Journal of *Molecular Sciences*

MDPI

Article—Supplementary Figures

Regulation of ER Composition and Extent, and Putative Action in Protein Networks by ER/NE protein TMEM147

Giannis Maimaris [1], Andri Christodoulou [1], Niovi Santama [1] and Carsten Werner Lederer [2,*]

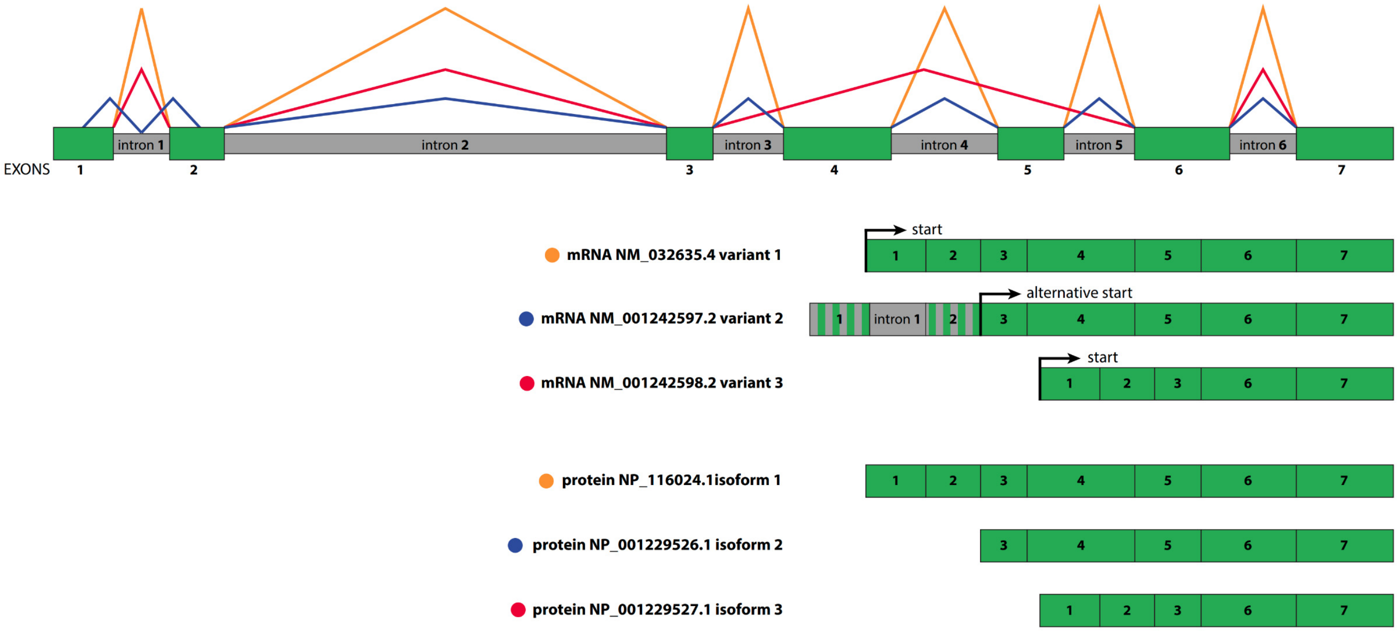

Supplementary Figure S1. The three main splice variants and isoforms of human TMEM147. Color codes (bright green, blue and red) indicate correspondence of splice events, transcripts and proteins.

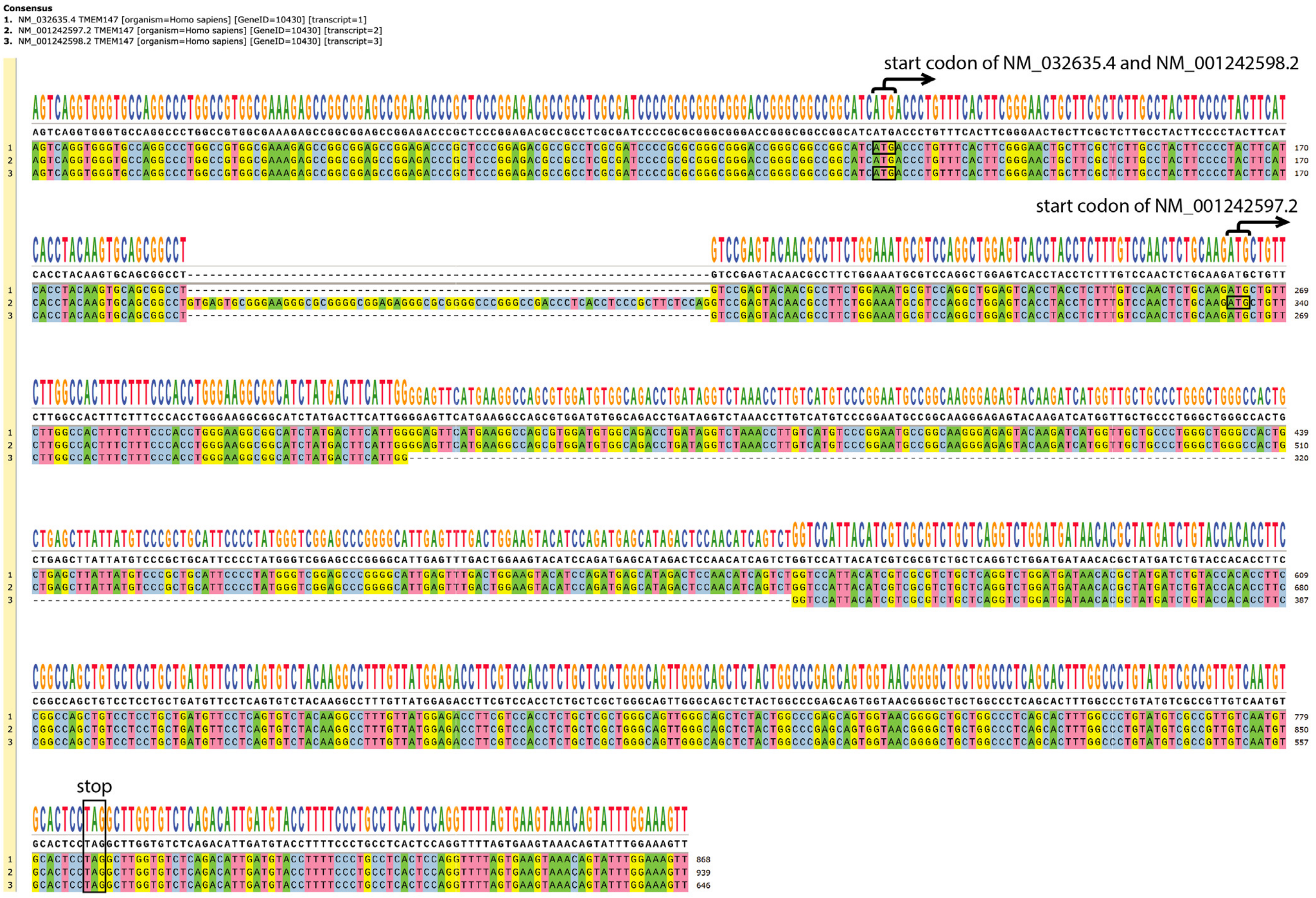

Supplemementary Figure S2. Alignment of RefSeq cDNA sequences for the three main TMEM splice variants

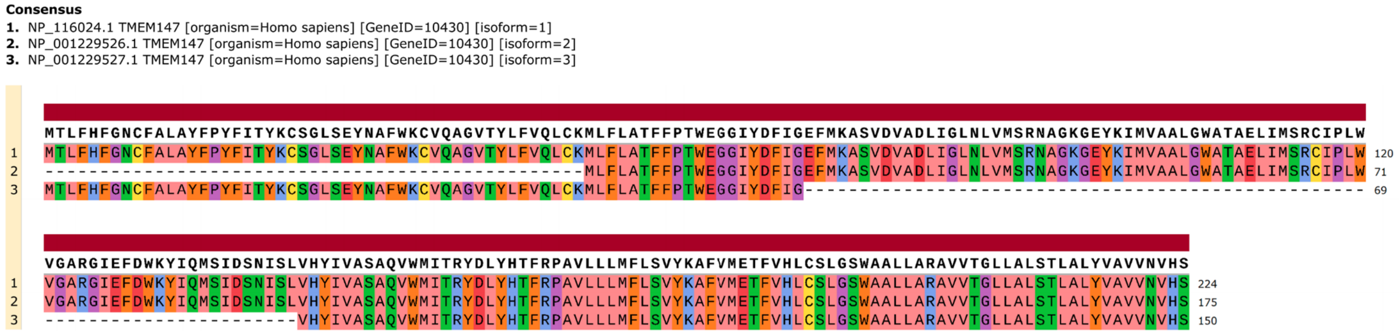

Supplementary Figure S3. Alignment of RefSeq protein sequences for the three main TMEM147 protein isoforms.

International Journal of *Molecular Sciences*

MDPI

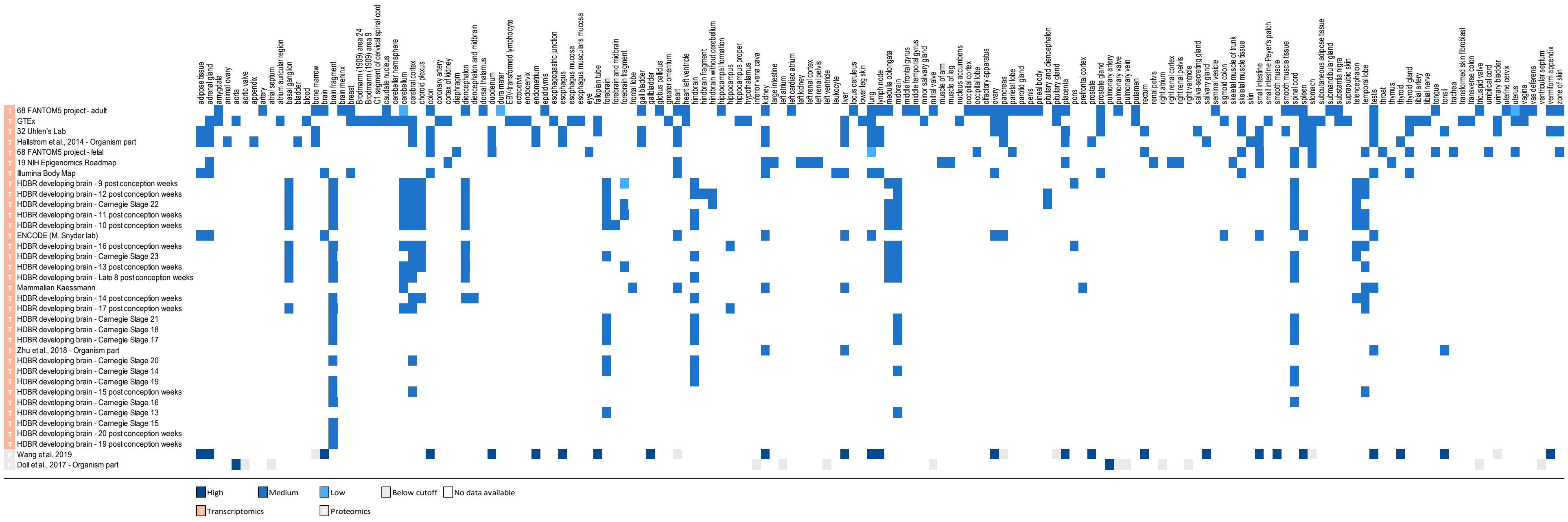

Supplementary Figure S4. Transcript and protein expression of TMEM147 across all isoforms. Expression data as extracted from https://www.ensembl.org/Homo_sapi-ens/Gene/ExpressionAtlas?db=core;g=ENSG00000105677;r=19:35545600-35547526 (28 August 2021) and reformatted in Microsoft Excel.

International Journal of
Molecular Sciences

MDPI

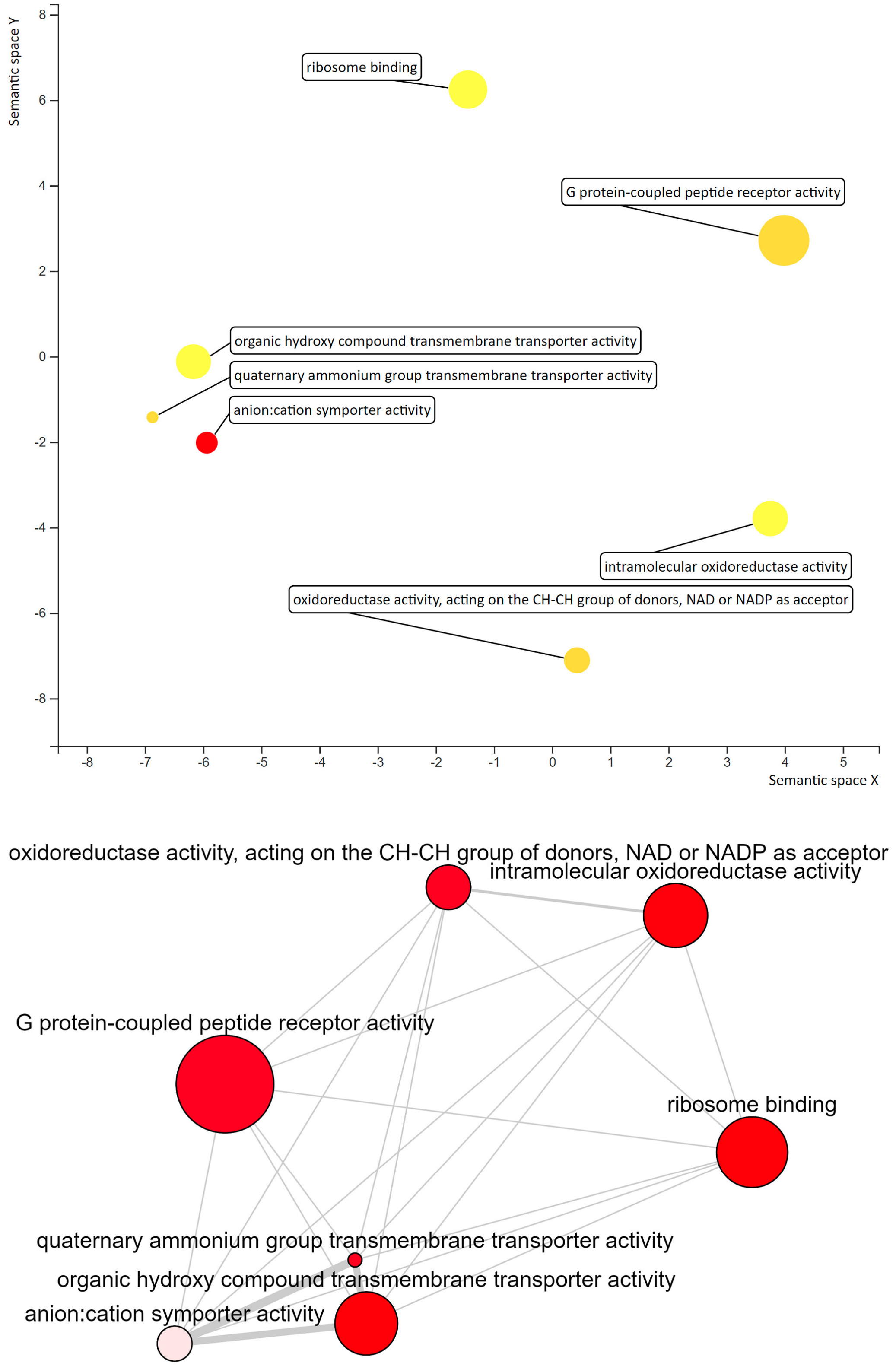

Supplementary Figure S5. Alternative simplified display of TMEM147-associated GO terms. In addition to TreeMaps, REVIGO provides a Scatterplot (top panel) and an Interactive Map (bottom panel) of terms. The Interactive Map is arranged for legibility and proximity of related terms, i.e. those with identical color code in the corresponding TreeMap.
